# Pneumococcal carriage among young children attending daycare in Hungary, 12–13 years post-PCV13: a cross-sectional study

**DOI:** 10.1038/s41598-025-07777-x

**Published:** 2025-07-02

**Authors:** Andrea Horváth, Annamária Huber, Árpád Bartha, Szofia Hajósi-Kalcakosz, Katalin Kristóf, Orsolya Dobay

**Affiliations:** 1 Institute of Medical Microbiology, Semmelweis University, Nagyvárad tér 4, Budapest, 1089 Hungary; 2 Department of Infectious Diseases, Heim Pál Children’s Hospital, Üllői út 86, Budapest, 1089 Hungary; 3 Institute of Laboratory Medicine, Semmelweis University, Üllői út 78/a, Budapest, 1082 Hungary

**Keywords:** Pneumococcus, Carriage, Serotypes, Antibiotic susceptibility, Vaccination, Risk factors, Microbiology, Bacteria, Vaccines

## Abstract

Nasopharyngeal carriage of *Streptococcus pneumoniae* in children plays a key role in transmission and disease epidemiology, hence it is crucial to monitor colonisation. As PCV-15 has recently replaced PCV-13 in Hungary, the aim of the study was to survey colonisation epidemiology 12–13 years post-PCV-13. 401 children, attending day care centres at Northern Hungary (including Budapest) were screened between April 2022 and April 2023. The samples were collected with cotton swabs from both nostrils, the serotype and antibiotic susceptibility of the isolates was determined. Overall, the carriage rate was 16.5% (66/401 children), which is lower compared to previous data from Hungary. Serotypes 23B, 35F, 15A/F, 15B/C, 11A and 23A were most prevalent. Non-vaccine types (NVTs) accounted for 68.2%, the coverage of PCV-13, PCV-15 and PCV-20 would be 9.1%, 10.6% and 31.8%, respectively. The resistance of the isolates to erythromycin, clindamycin and tetracycline was 12.1%, 7.6% and 10.6%, respectively and penicillin non-susceptibility was 24.2%. Serotypes 23B and 15A/F and 35F contributed mostly to resistance. In summary, prevalence of NVTs is increasing and these are associated with higher resistance rates. Among vaccine types, those included in PCV-20 are most prevalent. Hence, vaccination with the highest coverage could be recommended.

**Supplementary Information:**

The online version contains supplementary material available at 10.1038/s41598-025-07777-x.

## Introduction

*Streptococcus pneumoniae* (pneumococcus) can cause several mucosal (pneumonia, otitis media, sinusitis) and life-threatening invasive (sepsis, meningitis) infections, which occur mostly in young children and the elderly^1^. According to the WHO, this bacterium was still responsible for an estimated number of 300,000 pneumococcal disease associated deaths among children under five years world-wide in 2015, with most cases occurring in Africa and Asia^2^. Case fatality of childhood meningitis can be as high as 50%. Furthermore, a quarter of the survivors experience long-term neurological damages. Although mucosal infections are much less severe, they represent a significant health and economic burden worldwide.

Pneumococcal infections are transmitted by respiratory droplets, with asymptomatic carriers being the most frequent reservoirs. Pneumococci, being part of the normal microbiota viridans streptococci, are frequent colonisers of the human nasopharynx (NP). Carriage rate is age dependent. According to the large cohort study by Boegart et al., screening 3200 healthy Dutch children and adolescents aged 1–19 years, carriage peaks at around the age of 3 years with up to 55% prevalence and almost completely disappears by adulthood^3^. Carriage - as a precursor of disease - plays a determinative role in transmission dynamics, hence changes in carriage prevalence and serotype distribution as key epidemiological endpoints are used to assess vaccine impact and predict future disease trends.

As a prevention, vaccination was developed against this pathogen already in the 1910s, containing four serotypes and later six, but their use faded with the introduction of antibiotics. The first 14-valent vaccine was licensed in 1977 in the USA, followed by the currently still used 23-valent vaccine in 1983 (Pneumovax)^4^. The vaccines are based on the capsular polysaccharide (cps) of the bacterium, which represents its major surface antigen. Currently, there are two different types of vaccines: containing purified cps alone or chemically conjugated to a carrier protein (PCV, pneumococcal conjugated vaccine). Polysaccharides elicit a T-cell–independent immune response, fail to build immunological memory in small children, and have no effect on pneumococcal carriage. Hence, protein conjugation is particularly relevant when vaccinating young children^5^.

The PCVs have experienced several generations already. PCV-7 was licensed first in 2000 in the US, this was later followed by PCV-10 and PCV-13. Very recently, PCV-15 or Vaxneuvance (MSD) and PCV-20 or Prevenar 20 (Pfizer) have been authorised for use in the European Union by the European Medicines Agency (EMA) for both adults and children. The intended role of the new vaccines is addressing residual disease burden caused by the most prevalent invasive and virulent types. The additional serotypes in PCV-15 and PCV-20 are 22F, 33F and 8, 10A, 11A, 12F, 15B, respectively.

As the pneumococcus has to date 101 known different capsular serotypes^6^ and the vaccines contain only a proportion of it (10–23 types), vaccination represents a huge selection pressure on the species. All over the world, significant serotype re-arrangements were observed within a short period after the introduction of a new conjugate vaccine. Both the PCV7 and later the PCV13 have led to large scale epidemiological changes. The vaccine types (VT) disappeared or their occurrence decreased significantly, while non vaccine types (NVT) emerged and replaced these^7^. We can predict that this will occur also now, when the next generation PCVs will be used. It must be noted, however, that there are significant differences in invasiveness, transmissibility, and antibiotic resistance among these serotypes, and these factors are critical to evaluating the potential benefits of higher-valency vaccines.

In Hungary, PCV-7 vaccination was recommended for universal use in children in 2009; it was replaced by PCV-13 in 2010, which was then inserted in the Hungarian national immunisation programme as mandatory vaccination in July 2014 in a 2 + 1 scheme^8,9^. Children born after 30 September 2023 receive already PCV-15.

The Hungarian vaccination policy is very strict. From 2009 onwards, the vaccination rate has quickly jumped to > 80%^10^ and ever since then it has been reaching 99.9%, according to the annual vaccination reports^11^. Hungary is among those few countries (with Poland, Slovakia, Croatia, France, Bulgaria and Latvia in Europe), where pneumococcal vaccination is mandatory^12^, although WHO recommends the inclusion of PCVs in childhood immunization programmes worldwide^2^.

Examination of changes in colonization serotypes is particularly interesting, as the paediatric use of PCVs not only reduces vaccine types in children but, through herd immunity, influences also adult infections^5^. Furthermore, carried isolates can serve as reservoir for resistance and as a precursor to resistant disease-causing strains. Therefore, the aim of the current study was to conduct a cross-sectional analysis among asymptomatic carriers, 12–13 years after the introduction of PCV13 and before the anticipated serotype shifts following PCV15 implementation.

## Results and discussion

### Carriage rate

From the total of 401 screened children, 66 proved to be a pneumococcus carrier (age range 1–8 years). This equals to a 16.5% carriage rate (13.0–20.5 at 95% CI). None of the 40 adults (kindergartners) were colonised with pneumococci.

Initially, in the first few years after the introduction of the conjugate vaccines, no significant decrease in the carriage rate was to observe^13,14^, but there was already a strong re-arrangement in the serotypes. However, now, after several years of PCV implementation, we can witness a decrease also in the pneumococcal carriage rate. We obtained a much lower carriage rate (16.5%) in this study, compared to previous figures from Hungary, also screening children aged 3–6 years, attending day-care centres. We have conducted a survey immediately after PCV7 vaccination increased, in 2009–2012 (2262 children). In this period, the average carriage rate was 34.1%^13,15^. Later on, 6–7 years post-PCV7, after the introduction of PCV13, the carriage rate decreased to 21.5% in the same age category (580 children)^16^. This figure further decreased to 16.5% in the current study, i.e. 12–13 years post-PCV13 (Table 1).

**Table 1:** Decreasing trend in the carriage rate over the past 15 years in Hungary.

Screening period		Number of children	Carriage rate (%)	Reference
2009–2012	0–3 years post-PCV7	2262	34.1	^13,15^
2015–2016	6–7 years post-PCV7	580	21.5	^16^
2022–2023	13–14 years post-PCV7	401	16.5	this study

Other countries, where pneumococcal vaccination was introduced to the national immunization program, have reported similar tendency. A recent study from Turkey reported similarly low carriage rate (17.8%) among children < 5 years old, tested between March 2019 and March 2020, i.e. before the COVID pandemic^17^. In a large Chinese meta-analysis a significant decrease in carriage was shown as well, from 25.8% before the introduction of PCV7, to 14.1% in the post-PCV7 period^18^. Authors from Lima, Peru have also reported a significant decrease in carriage comparing the pre-PCV7 era (31.1%) with the post-PCV13 era (20.8%)^19^. Dutch authors have reported a decrease in carriage seven years after the introduction of PCV7 both in the 24 months age category and among the parents^20,21^.

### Risk factors for carriage

Carriage peaked in the 3-4y age category with 20.6%; younger or older children were colonised to lesser extent, but this was statistically significant only in the > 5 years age category (OR = 0.42, *p* = 0.04, Table 2). Although statistically not supported, but a weak positive trend toward carriage was observed in case of male gender (OR = 1.62, *p* = 0.07) and otitis media (OR = 1.78) or invasive infections (pneumonia or meningitis, OR = 1.61) in the medical anamnesis; whereas exposure to passive smoking and previous hospitalisation had slightly decreased the likelihood of carriage (OR = 0.55 and 0.66, respectively). Recent antibiotic treatment and having siblings had a negligible effect on carriage. Children living in the capital city versus in the country showed almost equal carriage rates (Table 2). Co-colonisation with other bacteria such as *Staphylococcus aureus*, *Haemophilus influenzae*, *Moraxella catarrhalis* might also influence pneumococcal carriage and vice versa^16^, but this was not investigated in this study.

**Table 2:** Univariate analysis of risk factors for Pneumococcus carriage in this study.

		*n*	% of all	Carriage rate *n* (%)	OR	95% CI	*p*
	All participants	401	100	66 (16.5)			
Variables							
Type of settlement	Capital city	255	63.6	40 (15.7)	1		
	Other	146	36.4	26 (17.8)	1.1646	0.68–2.0	0.5816
Gender	Female	198	49.4	26 (13.1)	1		
	Male	203	50.6	40 (19.7)	1.6234	0.95–2.78	0.0749
Age	< 3	103	25.7	18 (18.4)	0.8739	0.44–1.73	0.6991
	3–4	107	26.7	22 (20.6)	1		
	4–5	103	25.7	14 (13.6)	0.6006	0.29–1.25	0.1687
	> 5	80	20.0	8 (10.0)	0.4177	0.18–0.99	0.0395*
	No answer	8	2	3 (37.5)			
Siblings	No sibling	36	9	6 (16.7)	1		
	1–2 siblings	250	62.3	45 (18.0)	1.0976	0.43–2.79	0.8439
	≥ 3 siblings	20	5.0	4 (20)	1.2500	0.31–5.09	0.7565
	No answer	95	23.7	11 (11.6)			
Medical anamnesis	No otitis media	306	76.3	47 (15.4)	1		
	Otitis media	45	11.2	11 (24.5)	1.7829	0.84–3.77	0.1432
	No answer	50^1^	12.5				
	No pneumonia/meningitis	336	83.8	54 (16.0)	1		
	Pneumonia/meningitis	17	4.3	4 (23.5)	1.6068	0.51–5.11	0.4399
	No answer	48	12.0	8 (16.7)			
	No recent antibiotic treatment	304	75.8	51 (16.8)	1		
	Recent antibiotic treatment	47	11.7	7 (14.9)	0.8681	0.37–2.05	0.7435
	No answer	50	12.5	8 (16.0)			
	No previous hospitalisation	344	85.8	57 (16.6)	1		
	Previous hospitalisation	9	2.2	1 (11.1)	0.5529	0.07–4.61	0.5574
	No answer	48	12	8 (16.7)			
	No passive smoking	229	57.1	42 (18.3)	1		
	Passive smoking	124	30.9	16 (12.9)	0.6596	0.35–1.23	0.1813
	No answer	48	12.0	8 (16.7)			

*The medical anamnesis was not known for 48 children.

It has to be noted that only univariate analysis was applied instead of multivariable modelling. This might weaken the interpretation of potential determinants.

### Serotype distribution

**Fig. 1:**
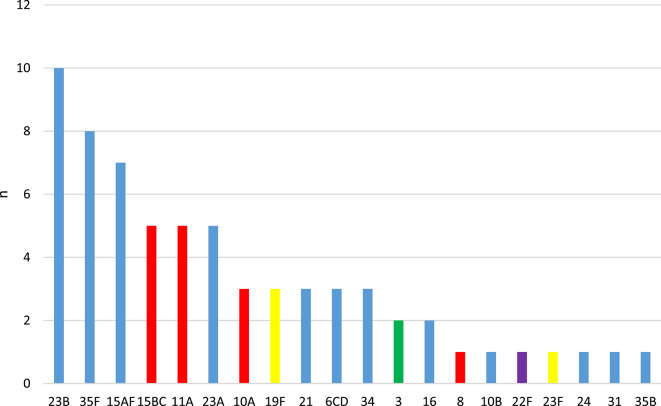
Serotype distribution of the 66 pneumococcal isolates.

The six most prevalent serotypes in ranking order were 23B, 35F, 15A/F, 15B/C, 11A and 23A (Fig. 1). Non-vaccine types (not included in any pneumococcal vaccine) were most prevalent, accounting for 68.2% in total. The PCV13 serotypes were represented only by six isolates (9.1%): 19F (*n* = 3), 3 (*n* = 2) and 23F (*n* = 1). The coverage of PCV15 and PCV20 would be 10.6% and 31.8%, respectively, over this strain collection (Table 3).

(yellow bars: PCV7 serotypes; green bar: PCV13 additional serotype; purple bar: additional PCV15 serotype; red bars: additional PCV20 serotypes; blue bars: NVT, not included in any pneumococcal vaccine)

**Table 3:** Coverage of the different conjugate vaccines on the carried isolates of this study (number and percentage of the serotypes belonging to each PCV).

	Serotypes																				*n*	%
PCV13	4	6B	9V	14	18C	19F	23F	1	5	7F	3	6A	19A									
	0	0	0	0	0	**3**	**1**	0	0	0	**2**	0	0								6	9.1
PCV15	4	6B	9V	14	18C	19F	23F	1	5	7F	3	6A	19A	22F	33F							
	0	0	0	0	0	**3**	**1**	0	0	0	**2**	0	0	**1**	0						7	10.6
PCV20	4	6B	9V	14	18C	19F	23F	1	5	7F	3	6A	19A	22F	33F	8	10A	11A	12F	15B		
	0	0	0	0	0	**3**	**1**	0	0	0	**2**	0	0	**1**	0	**1**	**3**	**5**	0	**5**	21	31.8

A similar serotype spectrum was found also by others. For instance, the ranking order of serotypes was 15B, 23F, 23A, 11A, 19F, 15F in the above mentioned Turkish study^17^; and 15B/C, 23B, 11A, 15A and 35B in a French study conducted in 2021–2022^22^. Candeias et al. reported 15B/C, 11A, 23B, 23A, 21 and 15A with the highest prevalence in Portugal in 2024^23^. Based on these data, more or less the same serotypes are circulating all across the European countries.

**Fig. 2:**
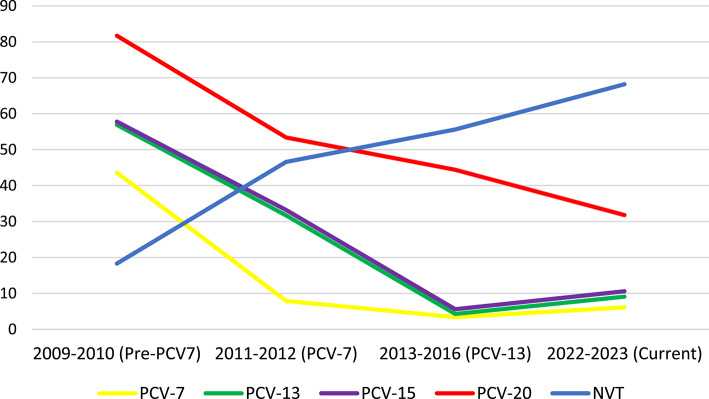
Changes in vaccine coverage among carried pneumococci over the different PCV periods in Hungary. *NVT* non-vaccine type.

Based on our results, the highest vaccine coverage is currently provided by PCV20, whereas that of PCV15 and PCV13 are comparable. The same was observed also in Turkey^17^. This has been the situation in Hungary since the implementation of PCV-7, which is attributable to the fact that we have always detected only very few strains belonging to the additional PCV-15 serotypes (22F, 33F), for instance in the current study only one strain of 22F. The potential coverage of the different vaccines has been declining continuously and in parallel to this, the NVTs are represented in an increasing ratio^13,15,16^ (Fig. 2).

However, it is important to note that different serotypes pose different invasive potential and suppression of the more invasive and virulent types should specifically be targeted by the new vaccines. Cohen et al. compared the serotype-specific prevalence of pneumococci in carriage versus in invasive isolates and they concluded that among non-PCV13 serotypes, 12F, 24F, 38, 8, 33F, 22F and 10A had the highest invasive potential^24^. Lindstrand et al. found that serotypes 12F, 7F, 8, 3, 9N and 22F were associated with the highest invasive potential, in the order of decreasing odds ratios^14^. Data on IPD in Europe is available from the ECDC Surveillance Atlas of Infectious Diseases^25^. According to this, serotypes 3, 8, 19A, 22F and 6C were most prevalent among IPD in Europe in 2022. Out of these, 22F and 33F are additional PCV-15 and PCV-20 serotypes (versus PCV-13) while 8, 10A and 1 F are exclusively PCV-20 serotypes.

On the other hand, if we look at the first seven most prevalent serotypes in our study, we find that these were either associated with low invasiveness (e.g. 23B, 35F–11A), or were equivalently important in both carriage and IPD (15A and 10A)^14,24,25^. It seems that although new serotypes keep replacing the disappearing vaccine types in colonisation, these novel serotypes are associated with decreased invasiveness.

In Fig. 2, we can see a minor increase in the PCV-7, PCV-13 and PCV-15 values in the last segment; this could be explained by the re-emerge of the PCV-7 serotype 19F.

Serotype 19F was represented by three isolates in this study, which corresponds to 4.5% (3/66). After a fluctuating prevalence of 19F in carriage over the preceding 10 years in Hungary, we have detected a highly similar figure to this in our last study in 2019 (4.9%)^26^. The persistence of 19F in colonisation was also experienced in other countries. For instance, in Portugal, whereas serotypes 3 and 19A were found mostly in unvaccinated children, 19F had a similar prevalence regardless the vaccination status^23^. According to a meta-analysis conducted about vaccine failure cases, 19F was found to be associated with the highest failure rate, with 39.8% out of 128 cases^27^. In Alaska, the re-emergence of 19F was documented among older children already in 2018 and was explained by the emergence of a novel genotype^28^. We have identified the MLST types of the three 19F isolates. One strain belonged to ST179, which was the dominant clone type among 19F strains in our previous screening study^26^, indicating the persistence of this clone among the Hungarian paediatric population. The other two 19F strains belonged to ST423, which was not observed in Hungary before and it is not related to any previously detected types. Further details about these two clones are provided in Supplementary File 1.

The use of pneumococcal conjugate vaccines in the frame of national immunisation programmes had a significant decreasing impact on IPD rates not only in the vaccinated paediatric population, but in non-vaccinated age cohort as well^29^. This indirect effect is mediated mostly by a reduction of vaccine-type nasopharyngeal carriage and thus transmission from vaccinated children to other susceptible individuals^30^. Wilson et al. have developed a model to predict the IPD rates over a future 7-year period in the Netherlands, if applying different vaccination strategies, and found that the best protection could be achieved if always the highest available valency vaccine was implemented^31^.

### Antibiotic susceptibility

None of our pneumococcal isolates were fully resistant to penicillin (Table 4), and 24.2% (n = 16) belonged to the „susceptible, increased exposure” category (I, MIC = 0.12-2 mg/L); these were serotypes 23B (*n* = 8), 15A/F (*n* = 3), 6 C/D, 15B/C, 23F, 34 and 35B. Comparable non-susceptibility figures were reported from Portugal (18.8%) and Spain (16%) as well^23,32^. Only the three isolates (one each of serotype 15A/F, 6C/D and 35B) with the highest penicillin MIC (= 2 mg/L) had elevated MICs to amoxicillin, cefotaxime and ceftriaxone. None of the isolates showed resistance to cephalosporins or imipenem (Table 4).

**Table 4:** Antibiotic susceptibility of the 66 Pneumococcal isolates from this study.

	MIC range (mg/L)	Susceptible, *n* (%)	Susceptible, increased exposure, *n* (%)	Resistant, *n* (%)
Penicillin	≤ 0.006–2	50 (75.8)	16 (24.2)	0 (0)
Amoxicillin	≤ 0.006–4	63 (95.5)	2 (3)	1 (1.5)
Cefotaxime	≤ 0.006–1	63 (95.5)	3 (4.5)	0 (0)
Ceftriaxone	≤ 0.006–1	65 (98.5)	1 (1.5)	0 (0)
Imipenem	≤ 0.003–0.25	66 (100)	0 (0)	0 (0)
Levofloxacin	≤ 0.5–1	0 (0)	66 (100)	0 (0)
Moxifloxacin	≤ 0.25	66 (100)	0 (0)	0 (0)
Erythromycin	≤ 0.125–>256	58 (87.9)	0 (0)	8 (12.1)
Clindamycin	0.125–>256	61 (92.4)	0 (0)	5 (7.6)
Linezolid	≤ 2	66 (100)	0 (0)	0 (0)
Vancomycin	≤ 1	66 (100)	0 (0)	0 (0)
Tetracycline	≤ 1–16	59 (89.4)	0 (0)	7 (10.6)
Rifampicin	≤ 0.25	66 (100)	0 (0)	0 (0)
Trimethoprim/sulfamethoxazole	≤ 0.5-8	61 (92.4)	2 (3.0)	3 (4.5)

Eight isolates were resistant to macrolides. Five of them (serotypes 15A/F, 19F and 6C/D) showed the MLSB type and three (serotypes 35 F and 34) had the M type. None of the isolates had inducible clindamycin resistance. Tetracycline resistance was present in case of seven isolates, with an MIC range of 2–24 mg/L. Only three isolates (= 4.5%) showed resistance to TMP/SMX, with MICs of 4–8 mg/L. None of the isolates were resistant to fluoroquinolones and all were fully susceptible to imipenem, moxifloxacin, linezolid, vancomycin and rifampicin. Among the 66 isolates, only three were multiresistant, i.e. resistant to three or more antibiotic classes (serotype 15A/F, *n* = 2 and 6C/D, *n* = 1).

Throughout the last 15 years, full penicillin resistance was virtually zero among the Hungarian carried isolates^13,15,16^. The macrolide resistance rate shows a significant decreasing tendency, whereas penicillin intermediate resistance rate decreased over the first seven years, but came back to 24.2% again. These changes could be explained by the changing serotype prevalence, especially that of 23B. Serotype 23B was absent in the earliest period, one single strain was observed in the PCV-7 period (0.2%), it had a prevalence of 7.4% in the PCV-13 period^13,15,16^, and became the leader in the current study with 15.2%. Most isolates of this type are intermediately resistant to penicillin (characteristic MIC = 0.25 mg/L), but fully susceptible to macrolides. As a consequence, penicillin intermediate resistance increased in the last period, but macrolide resistance remained low.

Different serotypes are associated with characteristic antibiotic resistance trends and even two types within the same serogroup can have different patterns (Supplementary Table 2). For instance, whereas many serotype 15A/F isolates had intermediate penicillin resistance, full macrolide and tetracycline resistance, only a single 15B/C isolate had elevated penicillin MIC. Within serogroup 23, most 23B isolates, as well as the single 23F isolate, had intermediate penicillin resistance (MIC = 0.25 mg/L) and non-susceptibility to TMP/SMX, while the 23A isolates were fully susceptible to all tested drugs. Out of the three 6C/D isolates, two were fully susceptible, but one had high-level resistance to macrolides and TMP/SMX and also elevated beta-lactam MICs. The serotype 19F isolates were all resistant to tetracycline.

In general, the serotypes mostly contributing to resistance were 23B, 15A/F, 35F and 19F. Out of these, the first three are non-vaccine types. Similar observation was published in 2022 by the CDC Active Bacterial Core surveillance, showing that the most prevalent serotypes contributing to multi-drug resistance among invasive pneumococcal disease (IPD) were 35B, 15A, 19A and 23A^33^. They observed that in parallel to the decline of IPD caused by vaccine-type non-susceptible pneumococci (VT-NS-IPD), the incidence of non-vaccine-type NS-IPD increased in all age groups, especially among the elderly (> 65y). This increase in resistant strains among the replacing NVTs is of worry and shouts for continuous monitoring.

### Comparison of carried and clinical isolates

**Fig. 3:**
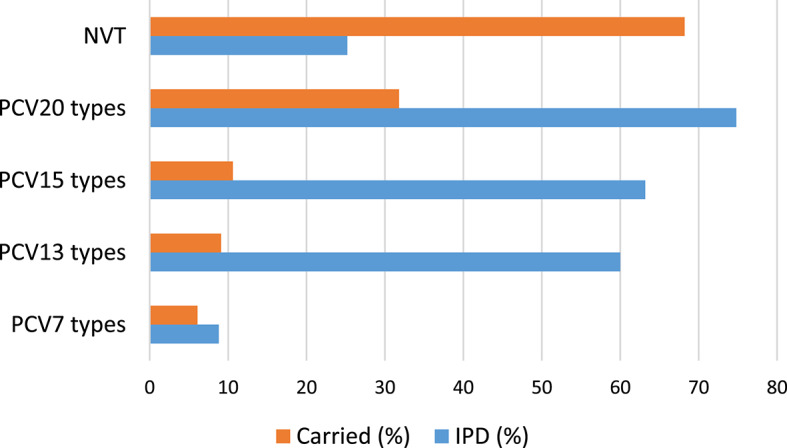
Vaccine coverage of the carried isolates of this study (2022–2023), compared to Hungarian invasive isolates (2023 data).

It is important to compare the serotype distribution of the carried and disease causing isolates. Information about Hungarian IPD isolates is managed by the National Center for Public Health and Pharmacy (NCPHP) in the frame of a passive surveillance system and the data are reported to ECDC annually. Serotyping data are displayed in the ECDC Surveillance Atlas of Infectious Diseases^25^, but only for selected years (e.g. 2018, 2020, 2023). However, serotyping data for all years between 2016 and 2023 were provided by Dr. Zsuzsanna Molnár, leader of the Department of Epidemiology and Infection Control at NCPHP (personal communication). According to these data, the overwhelming majority of invasive isolates belonged to serotype 3 (ranging from 25.7% in 2016 to 49.2% in 2022), followed by serotype 8 each year since 2017 (range 5.1-15.7%). The third position was taken variably by serotype 9N, 19A, 19F, 22F or 23B (range 4.3-7.9%). The calculated coverage of the conjugate vaccines among the Hungarian IPD isolates for the period 2016–2023 is the following: PCV-13: 44.9-60.0% (average = 54.5%), PCV-15: 47.5-63.2% (average = 58.0%), PCV-20: 61.6-77.1% (average = 72.8%). The much higher vaccine coverage among the IPD isolates (compared to carried isolates) can be explained by the very divergent serotype distribution in the two groups, especially the high prevalence of serotypes 3 and 8 in IPD (Fig. 3).

**Fig. 4:**
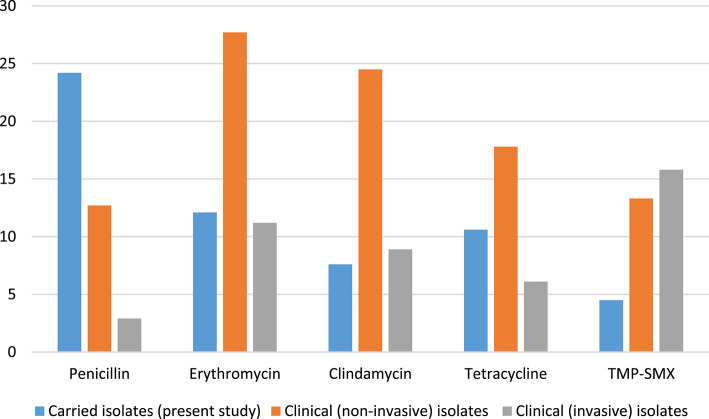
Antibiotic non-susceptibility rates of the carried pneumococci of this study, compared to Hungarian clinical isolates. *TMP-SMX* trimethoprim-sulfamethoxazole.

Antibiotic susceptibility data of Hungarian clinical pneumococcal isolates are reported annually by the NCPHP in the frame of the National Bacteriological Surveillance^34^. Comparing antibiotic susceptibility of clinical isolates from 2022 with the carried isolates reported in this study, the macrolide, tetracycline and TMP/SMX resistance is higher among disease causing (non-invasive) pneumococci, penicillin non-susceptibility is higher among the carried strains (Fig. 4). On the other hand, erythromycin and tetracycline resistance is somewhat lower in invasive isolates. This could be attributed again to the dissimilar serotype distribution among carried, invasive and non-invasive pneumococci. As serotype 3 is typically highly clonal and can be characterised with general susceptibility to antibiotics^26^, (although some resistant clones are emerging for example in the United States^35^), its dominant prevalence leads to overall reduced resistance rates among Hungarian IPD isolates.

As it was shown earlier, vaccine-driven serotype-rearrangement is seen earlier in colonisation and only with latency in clinical isolates^26^. Although we could detect divergent serotype and resistance profiles between the carried and clinical isolates, it is very important to monitor the epidemiological changes in both groups, to implement proper actions in prevention and treatment.

The limitations of the study are that (i) only a relatively low number of pneumococcal isolates were included in the examination due to the high reluctance of the parents to give permission for screening, (ii) the geographic scope of the study is limited as samples derived mostly from the Northern half of Hungary, (iii) only univariate (and not multivariate) analysis was applied in risk factor assessment, (iv) we could not detect multiple serotype carriage, as always 1–2 colonies from the primary culture were subcultured for further examinations, and (v) there is no available information on the serotypes of non-invasive clinical pneumococcal isolates.

## Conclusions

Taking the above mentioned limitations into consideration, according to our study, non-vaccine serotypes are dominant among carried pneumococci in Hungary, nonetheless, the coverage of PCV20 is still the highest (31.8%) among the available vaccines. The IPD isolates show a dissimilar serotype distribution with much higher PCV coverage, with serotype 8 being second most prevalent. However, monitoring serotype distribution in colonisation remains important, as these are reservoirs of disease.

Based on the above discussed scientific data obtained in this carriage study, as well as the data on Hungarian IPD serotypes, vaccination with the highest valency PCV (i.e., PCV20) should be considered in Hungary.

## Materials and methods

### The screened population

We have screened 401 children in total, between April 2022 and April 2023. The specimens were collected at 18 different day care centres / nurseries in Hungary. The majority derived from the capital city Budapest from 13 different institutions, the others from smaller cities and villages from Northern Hungary. Only those children were enrolled in the study, whose parents provided written informed consent on the child’s behalf. Further exclusion criterium was any acute respiratory illness. The gender of the participants was very much equalised: among the 401 children, 203 were males (50.6%) and 198 females (= 1,03:1 ratio). This represents the national demographic data well: in 2024, the male: female ratio in the 3-year old population was 1,05:1^36,37^. Furthermore, we have screened altogether 40 female kindergarten teachers, but their data was not included in further analysis. More than half of the children (53.4%) belonged to the 3–5 years age category, 26.2% were younger and 20.4% were older. It must be noted that the willingness of parents to give their consent for the screening was significantly lower compared to the pre-COVID era, very probably due to the unpleasant experiences with specimen collection for COVID testing. Out of the > 100 institutions approached, 18 agreed to participate in the survey. The number of the ethical permit for this study is TUKEB 4–5/2009, issued by the Regional and Institutional Committee of Science and Research Ethics of Semmelweis University, Budapest. All methods were performed in accordance with the relevant guidelines and regulations.

### Bacterial isolates

The samples were collected with sterile cotton swabs from the anterior parts of both nostrils, then transported to the laboratory within 24 h in active charcoal containing Amies transport gel (Transwab, Medical Wire & Equipment, Corsham, UK). The samples were inoculated on Columbia blood agar plates and incubated overnight at 37 °C in 5% CO_2_ atmosphere. The α-hemolytic colonies were subcultured and the purified pneumococci were tested for optochin sensitivity (5 µg discs, Mast Diagnostica, Bootle, UK) and their identity was confirmed with the PCR-based detection of the species-specific *lytA* gene^38^. All confirmed isolates were kept at − 80 °C on cryobeads until further testing.

### Serotyping

The serotype of the isolates was primarily determined by the Pneumotest Latex Kit (Statens Serum Institut, Copenhagen, Denmark). This kit contains 14 pooled antisera (A-I and P-T) with a checkerboard layout, designed for the identification of vaccine serotypes. For non-vaccine types and for the determination of the precise serotype within a serogroup (i.e., factor typing), PCR and factor sera were used. For the PCR, the primers were obtained from the CDC website^39^, published in the literature^40^, or designed by us^41^ (Supplementary Table 1). The PCR reactions were always performed individually. For the PCR, bacterial template DNA was prepared either by the boiling method, or by the ZR Fungal/Bacterial DNA MiniPrep purification kit (Zymo Research, Irvine, CA, US). On three occasions the serotyping result was uncertain, as the antisera and the PCR gave contradicting result. In these cases, the serotype was determined at the Institute of Laboratory Medicine of Semmelweis University (Budapest), by a Bruker MALDI Biotyper instrument (Beckton-Dickinson, Franklin Lakes, NJ, USA). This is a mass spectrometry based system, where isolates of the same serotype are arranged next to one another in the dendrogram, based on their unique proteomic fingerprint (i.e., the test isolates will be clustered with other isolates of known serotypes). These isolates proved to be serotype 15A/F (*n* = 1) and 23A (*n* = 2).

### Antibiotic susceptibility testing

The antibiotic susceptibility of the isolates was primarily determined by the VITEK 2 AST-P576 cards (bioMérieux, Marcy-l’Étoile, France), at the Institute of Laboratory Medicine of Semmelweis University (Budapest). The isolates were tested for 14 different antibiotics: penicillin, amoxicillin, cefotaxime, ceftriaxone, imipenem, levofloxacin, moxifloxacin, erythromycin, clindamycin, linezolid, vancomycin, tetracycline, rifampicin and trimethoprim/sulfamethoxazole (TMP/SMX). This method applies microdilution to determine the MIC in a narrow concentration range around the resistance breakpoint specific for pneumococci. In case of penicillin, erythromycin or tetracycline resistance, the strains were additionally tested with a gradient MIC test strip (Liofilchem, Roseto degli Abruzzi, Italy), over a wider dilution range. If an isolate was resistant to erythromycin, but sensitive to clindamycin, the D-test was also performed to check for inducible clindamycin resistance. The antibiotic susceptibility results were evaluated according to the EUCAST clinical breakpoints version 14.0 (2024)^42^, using the ATCC 49619 control strain.

### MLST

For MLST typing of the three serotype 19F isolates, seven housekeeping genes were amplified and sequenced using the Sanger method at Eurofins BIOMI Ltd, Gödöllő, Hungary. The allelic sequences were assigned to sequence types according to the pneumococcal MLST database available at http://pubmlst.org/spneumoniae/^43^.

### Statistical analysis

The association between potential risk factors and the risk of colonisation was studied using a univariate logistic regression model estimating unadjusted, crude odds ratios (ORs) and 95% CIs. Risk factors were considered significantly associated with carriage if p value was < 0.05. Statistical analyses were performed using MedCalc for Windows, version 22.014 (MedCalc Software, Ostend, Belgium).

Sample size calculation was based on Cochran formula, with 95% confidence interval (α = 0.05) and power of 80%. Estimating 20% carriage based on literature data and using the official Hungarian demographic data (*n* = 290286 children belonged to the 1–6 years age category in 2023^36,37^, 246 participants would be required for a representative sample size. Instead, we have screened 401 children.

## Electronic supplementary material

Below is the link to the electronic supplementary material.

Supplementary Material 1

Supplementary Material 2

Supplementary Material 3

## Data Availability

The datasets generated and/or analysed during the current study (MLST allele sequences of the three 19F isolates) are available in the MLST isolate collection, accessible at: https://pubmlst.org/bigsdb?db=pubmlst_spneumoniae_isolates. Reference numbers: 257391, 257392, 257393.
