# Supplementary material for: Pneumococcal carriage among young children attending daycare in Hungary, 12–13 years post-PCV13: a cross-sectional study
Source: Sci Rep. 2025 Jul 2;15:22696. doi: 10.1038/s41598-025-07777-x (PMC12215634; doi:10.1038/s41598-025-07777-x)
Supplement: Supplementary file 1 — Supplementary Material 1 [file 41598_2025_7777_MOESM1_ESM.docx]

**Supplementary file 2.** Description of ST423 and ST179 isolates (serotype 19F)

There are currently 90 ST423 pneumococci in the MLST database [42], all but one isolated in European countries, mostly from the Czech Republic, Poland and Germany, i.e. countries very close to Hungary, and the majority derive from blood or cerebrospinal fluid. This type was also reported from Portugal [23]. The two ST423 strains in our study were only resistant to tetracycline.

Regarding ST179, there are 127 records in the MLST database, several of which are from other continents (South America, Africa, Asia) and many isolates derive from otitis media or carriage, indicating the lesser invasive potential of this type. ST179-19F was also detected in Spain and Portugal recently [23, 32]. These strains are characterised with MLSB type macrolide resistance and tetracycline resistance, but full susceptibility to penicillin. Of note, two ST179 isolates were serotype 1 and one was serotype 23A; and two ST423 isolates were serotype 19A and one was serotype 9N, indicating the high likelihood of pneumococci for serotype switching.
