# Supplementary material for: Pneumococcal carriage among young children attending daycare in Hungary, 12–13 years post-PCV13: a cross-sectional study
Source: Sci Rep. 2025 Jul 2;15:22696. doi: 10.1038/s41598-025-07777-x (PMC12215634; doi:10.1038/s41598-025-07777-x)
Supplement: Supplementary file 2 — Supplementary Material 2 [file 41598_2025_7777_MOESM2_ESM.docx]

| **Supplementary Table 1.** Serotyping primers used in this study | | | | | | |  |
| --- | --- | --- | --- | --- | --- | --- | --- |
| Serotypes and primers | | | Sequence (5’→3’) | Amplicon size (bp) | Ref. | |  |
| 1 | for | GGAGACTACTAAATTGTAATACTAACACAGCG | | 99 | | [40] | |
|  | rev | CAAGGATGAATAAAGTAAACATATAATCTC | |  |  |  |  |
| 2 | for | TATCCCAGTTCAATATTTCTCCACTACACC | | 290 | | CDC [39] | |
|  | rev | ACACAAAATATAGGCAGAGAGAGACTACT | |  |  |  |  |
| 3 | for | TTGTTTTTTGTCTTTATTCTTATTCGTTGG | | 818 | | [40] | |
|  | rev | TTGTTTTTTGTCTTTATTCTTATTCGTTGG | |  |  |  |  |
| 4 | for | CTGTTACTTGTTCTGGACTCTCGTTAATTGG | | 430 | | [40] | |
|  | rev | GCCCACTCCTGTTAAAATCCTACCCGCATTG | |  |  |  |  |
| 5 | for | GAGACGTCTTTGGGGCATAA | | 366 | | own design | |
|  | rev | GCGGAAACGATGAGAAGAAG | |  |  |  |  |
| 6 A/B/C/D | for | CGACGTAACAAAGAACTAGGTGCTGAAAC | | 220 | | [40] | |
|  | rev | AAGTATATAACCACGCTGTAAAACTCTGAC | |  |  |  |  |
| 6 C/D | for | CATTTTAGTGAAGTTGGCGGTGGAGTT | | 727 | | CDC [39] | |
|  | rev | AGCTTCGAAGCCCATACTCTTCAATTA | |  |  |  |  |
| 7C/B/40 | for | CTATCTCAGTCATCTATTGTTAAAGTTTACGACGGGA | | 260 | | CDC [39] | |
|  | rev | GAACATAGATGTTGAGACATCTTTTGTAATTTC | |  |  |  |  |
| 7F/A | for | TGACTGCAAGTGTTTCAATGG | | 528 | | own design | |
|  | rev | CGTTTCCAAAAATTCCTCCA | |  |  |  |  |
| 8 | for | GAAGAAACGAAACTGTCAGAGCATTTACAT | | 201 | | CDC [39] | |
|  | rev | CTATAGATACTAGTAGAGCTGTTCTAGTCT | |  |  |  |  |
| 9 N/L | for | GAACTGAATAAGTCAGATTTAATCAGC | | 516 | | CDC [39] | |
|  | rev | ACCAAGATCTGACGGGCTAATCAAT | |  |  |  |  |
| 9V | for | AGAGGAGTTCAATCGCCAGA | | 242 | | own design | |
|  | rev | ATCGGTTCCCCAAGATTTTC | |  |  |  |  |
| 10A | for | GGTGTAGATTTACCATTAGTGTCGGCAGAC | | 628 | | CDC [39] | |
|  | rev | GAATTTCTTCTTTAAGATTCGGATATTTCTC | |  |  |  |  |
| 10F/10C/33C | for | GGAGTTTATCGGTAGTGCTCATTTTAGCA | | 248 | | CDC [39] | |
|  | rev | CTAACAAATTCGCAACACGAGGCAACA | |  |  |  |  |
| 12/44/46 | for | GCAACAAACGGCGTGAAAGTAGTTG | | 376 | | CDC [39] | |
|  | rev | CAAGATGAATATCACTACCAATAACAAAAC | |  |  |  |  |
| 11A/F/D | for | CGAAATATCGCCATTCATCA | | 379 | | own design | |
|  | rev | TCAACAGCAACTGTGCCACT | |  |  |  |  |
| 13 | for | ACGACTTGGAAGTGCTGCTT | | 308 | | own design | |
|  | rev | CCAAAAACAAAATCGCTGGA | |  |  |  |  |
| 14 | for | GTCTGTTTATTCTATATACAAAGAGGCTCC | | 268 | | [40] | |
|  | rev | GCATTGCTACAATCGCTATACTAGATATGC | |  |  |  |  |
| 15A/F | for | CATTTGCACCCTGACTTCAC | | 409 | | own design | |
|  | rev | GTCCCGCAAACTCTGTCCTA | |  |  |  |  |
| 15B/C | for | TGTTCAAAGAGGCGCTAATG | | 493 | | own design | |
|  | rev | TGTTCTGATTCCTGCTCCAA | |  |  |  |  |
| 16F | for | GAATTTTTCAGGCGTGGGTGTTAAAAG | | 717 | | CDC [39] | |
|  | rev | CAGCATATAGCACCGCTAAGCAAATA | |  |  |  |  |
| “18”  18 CFBA | for | CTTAATAGCTCTCATTATTCTTTTTTTAAGCC | | 573 | | CDC [39] | |
|  | rev | TTATCTGTAAACCATATCAGCATCTGAAAC | |  |  |  |  |
| “18C”  18 CFBA | for | GCCGTGGGAAGCTTATTTTT | | 285 | | own design | |
|  | rev | CCTGCCTAAAGGCAACAATG | |  |  |  |  |
| 19A | for | GTTAGTCCTGTTTTAGATTTATTTGGTGATGT | | 478 | | [40] | |
|  | rev | GAGCAGTCAATAAGATGAGACGATAGTTAG | |  |  |  |  |
| 19F | for | CACCTAATTTTAATACTGAGGTTAAGATTGC | | 408 | | [40] | |
|  | rev | CATAGGCTATCAGAATTTTAATAATATCTTGC | |  |  |  |  |
| 20 | for | ATCAGGAATACGCCAATCAA | | 195 | | own design | |
|  | rev | ATCGGTAATGCAAAGCCAAC | |  |  |  |  |
| 21 | for | CTATGGTTATTTCAACTCAATCGTCACC | | 192 | | CDC [39] | |
|  | rev | GGCAAACTCAGACATAGTATAGCATAG | |  |  |  |  |
| 22 F/A | for | GAGTATAGCCAGATTATGGCAGTTTTATTGTC | | 643 | | CDC [39] | |
|  | rev | CTCCAGCACTTGCGCTGGAAACAACAGACAAC | |  |  |  |  |
| 23A | for | GATTTGGAGCGGATCGATTA | | 823 | | CDC [39] | |
|  | rev | AATGGGTAATGGAGGGGAGT | |  |  |  |  |
| 23B | for | CCACAATTAGCGCTATATTCATTCAATCG | | 199 | | CDC [39] | |
|  | rev | GTCCACGCTGAATAAAATGAAGCTCCG | |  |  |  |  |
| 23F | for | GTAACAGTTGCTGTAGAGGGAATTGGCTTTTC | | 384 | | [40] | |
|  | rev | CACAACACCTAACACACGATGGCTATATGATTC | |  |  |  |  |
| 24A/B/F | for | GCTCCCTGCTATTGTAATCTTTAAAGAG | | 99 | | CDC [39] | |
|  | rev | GTGTCTTTTATTGACTTTATCATAGGTCGG | |  |  |  |  |
| 25F/25A/38 | for | CGTTCTTTTATCTCACTGTATAGTATCTTTATG | | 574 | | CDC [39] | |
|  | rev | ATGTTTGAATTAAAGCTAACGTAACAATCC | |  |  |  |  |
| 28 F/A | for | ACGGTCAGAGTTTGGTCGAG | | 339 | | own design | |
|  | rev | GAAAACGTGATTTCCGTCGT | |  |  |  |  |
| 31 | for | GGAAGTTTTCAAGGATATGATAGTGGTGGTGC | | 701 | | CDC [39] | |
|  | rev | CCGAATAATATATTCAATATATTCCTACTC | |  |  |  |  |
| 33F | for | TCCCCAACGGTTTATGTGTT | | 171 | | own design | |
|  | rev | CAATGCAAGGCTCAATACCA | |  |  |  |  |
| 34 | for | GCTTTTGTAAGAGGAGATTATTTTCACCCAAC | | 408 | | CDC [39] | |
|  | rev | CAATCCGACTAAGTCTTCAGTAAAAAACTTTAC | |  |  |  |  |
| 35B | for | GATAAGTCTGTTGTGGAGACTTAAAAAGAATG | | 677 | | CDC [39] | |
|  | rev | CTTTCCAGATAATTACAGGTATTCCTGAAGCAAG | |  |  |  |  |
| 35F/47F | for | GAACATAGTCGCTATTGTATTTTATTTAAAGCAA | | 517 | | CDC [39] | |
|  | rev | GACTAGGAGCATTATTCCTAGAGCGAGTAAACC | |  |  |  |  |
| 39 | for | TCATTGTATTAACCCTATGCTTTATTGGTG | | 98 | | CDC [39] | |
|  | rev | GAGTATCTCCATTGTATTGAAATCTACCAA | |  |  |  |  |
| 42/35A/  35C | for | TCCCTTTTTCAGACGTAGCC | | 492 | | own design | |
|  | rev | CAAGAAATTGATCCGCTTGGT | |  |  |  |  |
