# Supplementary material for: Pneumococcal carriage among young children attending daycare in Hungary, 12–13 years post-PCV13: a cross-sectional study
Source: Sci Rep. 2025 Jul 2;15:22696. doi: 10.1038/s41598-025-07777-x (PMC12215634; doi:10.1038/s41598-025-07777-x)
Supplement: Supplementary file 3 — Supplementary Material 3 [file 41598_2025_7777_MOESM3_ESM.docx]

**Supplementary Table 2**. Serotype-specific antibiotic resistance and multidrug resistance

| **Sero-type** | **23B** | **35F** | **15AF** | **15BC** | **11A** | **23A** | **10A** | **19F** | **21** | **6CD** | **34** | **3** | **16** | **8** | **10B** | **22F** | **23F** | **24** | **31** | **35B** | **Total (n(%))** |
| --- | --- | --- | --- | --- | --- | --- | --- | --- | --- | --- | --- | --- | --- | --- | --- | --- | --- | --- | --- | --- | --- |
| **n** | 10 | 8 | 7 | 5 | 5 | 5 | 3 | 3 | 3 | 3 | 3 | 2 | 2 | 1 | 1 | 1 | 1 | 1 | 1 | 1 | 66 |
| **AMR** | 8 | 2 | 3 | 1 | 1 | 0 | 0 | 3 | 0 | 1 | 1 | 0 | 0 | 0 | 0 | 0 | 1 | 1 | 0 | 1 | 23 (34.8) |
| **MDR** | 0 | 0 | 2 | 0 | 0 | 0 | 0 | 0 | 0 | 1 | 0 | 0 | 0 | 0 | 0 | 0 | 0 | 0 | 0 | 0 | 3 (4.6) |

AMR: non-susceptibility to at least one antibiotic

MDR: multi-drug resistance; non-susceptibility to three antibiotics of different classes
